# The Interplay between the Endocannabinoid System, Epilepsy and Cannabinoids

**DOI:** 10.3390/ijms20236079

**Published:** 2019-12-02

**Authors:** Keith A. Kwan Cheung, Hassendrini Peiris, Geoffrey Wallace, Olivia J. Holland, Murray D. Mitchell

**Affiliations:** 1 Institute of Health and Biomedical Innovation (IHBI), Faculty of Health, Queensland University of Technology (QUT), Centre for Children’s Health Research (CCHR), 62 Graham Street, South Brisbane, Queensland 4101, Australia; k.kwancheung@hdr.qut.edu.au (K.A.K.C.); hassendrini.peiris@qut.edu.au (H.P.); holland2@qut.edu.au (O.J.H.); 2 Children’s Health Queensland (CHQ) and University of Queensland (UQ), Centre for Children’s Health Research, 62 Graham Street, South Brisbane, Queensland 4101, Australia; Geoff.Wallace@health.qld.gov.au; 3 School of Medical Science, Griffith University, 1 Parklands Dr, Southport, Queensland 4215, Australia

**Keywords:** endocannabinoids, endocannabinoid system, epilepsy, neurological diseases, cannabinoids, cannabis, neuroinflammation, biomarkers

## Abstract

Epilepsy is a neurological disorder that affects approximately 50 million people worldwide. There is currently no definitive epilepsy cure. However, in recent years, medicinal cannabis has been successfully trialed as an effective treatment for managing epileptic symptoms, but whose mechanisms of action are largely unknown. Lately, there has been a focus on neuroinflammation as an important factor in the pathology of many epileptic disorders. In this literature review, we consider the links that have been identified between epilepsy, neuroinflammation, the endocannabinoid system (ECS), and how cannabinoids may be potent alternatives to more conventional pharmacological therapies. We review the research that demonstrates how the ECS can contribute to neuroinflammation, and could therefore be modulated by cannabinoids to potentially reduce the incidence and severity of seizures. In particular, the cannabinoid cannabidiol has been reported to have anti-convulsant and anti-inflammatory properties, and it shows promise for epilepsy treatment. There are a multitude of signaling pathways that involve endocannabinoids, eicosanoids, and associated receptors by which cannabinoids could potentially exert their therapeutic effects. Further research is needed to better characterize these pathways, and consequently improve the application and regulation of medicinal cannabis.

## 1. Introduction

Neurological disorders, such as epilepsy, tic disorders, dementia, multiple sclerosis, and Parkinson’s disease have constituted over 6% of the global disease burden since 2006, but gaps in our understanding of fundamental aspects of neurological disorders remain despite their broad health implications [1]. Recent research advances, including novel therapies, are helping to better elucidate the causes of neurological disorders, and to improve treatment of patients. In particular, the endocannabinoid system (ECS) is central to neurological function, and the modulation of this system shows therapeutic promise.

This review summarizes the current state of knowledge on the role of the ECS in the important neurological disorder of epilepsy and its interactions with cannabinoids in the context of epilepsy treatment. In particular, we consider the links between neuroinflammation, the ECS and cannabinoids.

Neuroinflammation has been detected in brains that have experienced epilepsy, which suggests a role in disease pathology. Specifically, neuroinflammation has been shown to occur both after an epileptic event and before the onset of epilepsy, which indicates that neuroinflammation might have a causal role in seizures.

We focus on the use of cannabis-based treatments that affect the ECS, how these treatments are particularly applicable in minors, and how the development of efficient biomarkers might be key to the effective use of these treatments. Cannabis-based treatments may provide both better management of epilepsy and improved understanding of ECS function in health and disease, but these treatments require more rigorous scientific evidence to validate their safety and fine-tune their use.

## 2. Epilepsy and Neuroinflammation

Epilepsy is the most common serious neurological disorder, and it is estimated to affect over 50 million people worldwide [2]. Epilepsy is a chronic disease of the brain that is characterized by recurrent seizures (at least two or more unprovoked seizures), which are brief episodes of involuntary movement or altered sensation that may involve a part of the body (focal) or the entire body (generalized). Seizure episodes are a result of excessive electrical discharges, particularly in the neurons, i.e., neuronal hyperexcitability. Different parts of the brain can be the site of such discharges; as such, some types of epilepsy tend to be named after the most affected site, e.g., temporal lobe epilepsy. Seizures can vary from the briefest lapses of attention or muscle jerks to severe and prolonged convulsions. Seizures can also vary in frequency, from less than one per year to several per day [2]. Refractory epilepsy is epilepsy resistant to standard pharmaceutical treatments. Individuals are diagnosed with refractory epilepsy when they are subject to epileptic seizures that are poorly responsive to the currently available treatment options (e.g., antiepileptic drugs (AEDs), ketogenic diets, high doses of steroids, and neurostimulation therapies) [3].

Epilepsy might occur as a result of neurological insults, deficits, or brain abnormalities, including traumatic, post-infection, neoplastic, developmental, and vascular lesions. However, only 20–30% of epilepsy cases have a known/suspected cause. The remaining 70–80% of epilepsies arise in the absence of obvious neurological deficits, intellectual disability, or brain injuries. Genetic factors are theorized to play a key etiological role in these idiopathic epilepsies [4,5]. Current treatments focus on the elimination or attenuation of seizures and their frequencies due to the broad range of potential epilepsy causes. Recently, intriguing links between epilepsy and neuroinflammation have been reported, whereby neuroinflammation is suggested to be a major pathophysiological mechanism directly related to the incidence and severity of epileptic seizures. The degree of neuroinflammation varies between different types of epilepsy and between patients. Therefore, a better understanding of the signaling pathways of neuroinflammation might open novel avenues for targeted treatment of epileptic seizures.

### The Role of the Endocannabinoid System in Neuroinflammation and Epilepsy

Epilepsy has been associated with ECS dysfunction and, in particular, neuroinflammation. There are already comprehensive reviews [6,7] detailing neuroinflammation and epilepsy, therefore this review will only briefly introduce this complex topic here. Neuroinflammation is a physiological response to insult and/or injury that is mainly mediated by glial cells in the brain. Microglia are a type of glial cells that serve as the resident immune cells of the central nervous system (CNS) and primarily function in protecting the neuronal population. The microglia are activated by pathogens, products from injured/inflamed neurons, and blood-brain barrier disruptions, alongside a wide variety of chemical threat signals, including chemokines and cytokines (e.g., Interleukin-1β (IL-1β), tumor necrosis factor-α (TNF-α)) [8,9,10]. While neuroinflammation is a normal defensive mechanism, it seems that there is an overreaction and inability to effectively downregulate the response in diseased brains, when compared to healthy brains [11]. Many of the ligands and receptors of the ECS are involved in inflammatory pathways. Therefore, the ECS is likely to be central in the development and occurrence of neuropathology, where the complex signaling pathways can be affected at multiple points.

There are already recent review articles detailing the ECS [12,13], so this broad topic will only be summarized here. The ECS is one of the major axes of the CNS. Its principal role is to modulate synaptic activity (excitatory and inhibitory) through the release of endogenous cannabinoids (endocannabinoids); this complex system is comprised of endocannabinoids, cannabinoid receptors, and the enzymes that are responsible for the synthesis and degradation of endocannabinoids [14]. The ECS is heavily involved in the regulation of several aspects of brain development/health, namely neural progenitor proliferation, lineage commitment, neuronal migration, axonal guidance, and synaptic plasticity [15]. Endocannabinoids (the two most studied being N-arachidonoyl-ethanolamine (anandamide or AEA) and 2-arachidonoylglycerol (2-AG)) are lipid-derived molecules that travel from the post- to the pre-synaptic site, where they bind to membrane-bound, cannabinoid receptors to modulate synaptic transmission via a process known as retrograde synaptic signaling (i.e., endocannabinoids enable neurons to influence the strength of their own synaptic inputs in an activity-dependent manner) [16]. The cannabinoid receptors are classified as G-protein coupled receptors (GPRs), being located throughout the entire human body–the receptors that we will discuss here are the most well characterized, i.e., the CB1, CB2, and GPR55 receptors. CB1 receptors (CB1R) are abundant in the CNS, particularly in the cortex, basal ganglia, hippocampus, and cerebellum; the majority of CB1 receptors are present on axon terminals and pre-terminal axon segments [14,17]. CB2 receptors (CB2R) are expressed at much lower levels in the CNS when compared to CB1R; this receptor is primarily present in microglia, vascular elements, immune cells, and some specific neurons [14,18]. GPR55 receptors are mostly located in the brain and peripheral nervous system [19,20], where their activation has been found to increase intracellular calcium release in neurons, which can lead to an increase in neuronal excitability [21].

**Figure 1:**
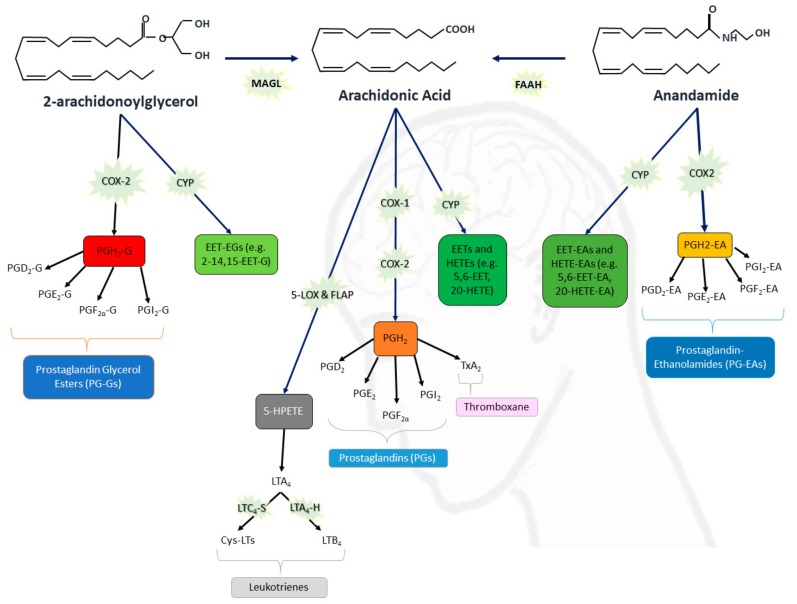
Eicosanoid and endocannabinoid pathways. Arachidonic acid (AA) can be synthesized from 2-arachidonoylglycerol (2-AG) and N-arachidonoyl-ethanolamine (anandamide or AEA) by monoacylglycerol lipase (MAGL) and fatty acid amide hydrolase (FAAH) respectively. Cyclooxygenases (COX-1 and COX-2) enable the conversion of 2-AG, AA and AEA into PGH_2_-Gs (prostaglandin H_2_-glycerol ester), PGH_2_ (prostaglandin H_2_), and PGH_2_-EA (prostaglandin H_2_-ethanolamide) respectively; the various enzyme systems that then convert these precursor molecules into prostanoids (e.g., PGE_2_-EA) are not shown in this diagram. Five-lipoxygenase activating protein (FLAP) enables 5-LOX enzyme to convert AA into 5-hydroperoxyeicosatetraenoic acid (5-HPETE) that rapidly dehydrates to form leukotriene A4 (LTA_4_). LTA_4_ is then converted into LTC_4_ by LTC_4_-synthase (LTC_4_-S) and into LTB_4_ by LTA_4_-hydrolase (LTA_4_-H). LTC_4_ can be further converted into additional cysteinyl-leukotrienes (Cys-LTs) LTD_4_ and LTE_4_ by other enzymes not depicted in this diagram [27]. A variety of cytochrome P450 (CYP) epoxygenases and hydrolases that are detailed in other reviews [28,29] catalyze the conversion of the endocannabinoids into epoxyeicosatrienoic acids (EETs), hydroxyeicosatetraenoic acids (HETEs), 2-11,12-epoxyeicosatrienoic glycerol (EET-Gs), epoxyeicosatrienoic acid-ethanolamides (EET-EAs), and hydroxyeicosatetraenoic acid-ethanolamides (HETE-EAs).

Activation/inhibition of the ECS receptors can modulate a wide range of intracellular and intercellular signaling activity, such as ion channels (i.e., potassium, sodium, and calcium channels), intracellular calcium ion concentrations, and inflammation [22]. Impaired endocannabinoid signaling might be crucial in epileptogenesis, as expressions of CB1R and diacylglycerol lipase-α (DAGL-α is one of the enzymes that synthesize 2-AG from polyunsaturated fatty acids) are reportedly downregulated in epileptic human hippocampi [23], while the levels of AEA are diminished in the cerebrospinal fluid of epileptic patients [24]. 2-AG is a full CB1R agonist while AEA is a partial CB1R agonist, and both endocannabinoids are GPR55 agonists [13,19,25]. AEA is mostly catalyzed to arachidonic acid (AA) and ethanolamine by the fatty acid amide hydrolase (FAAH) enzyme at the end of the endocannabinoids’ lifecycles (illustrated in Figure 1) [22], while 2-AG is mostly converted to AA and glycerol by monoacylglycerol lipase (MAGL) [26].

The cyclooxygenase enzyme (COX) catalyzes the first step in the synthesis of lipid-derived signalling mediators, known as prostanoids, often by using AA as a substrate, and these pathways can be proinflammatory. COX exists in two homologous isoforms. The constitutive isoform, COX-1, which is widely distributed in various cell types, is thought to mediate physiological responses, while the inducible isoform, COX-2, is rapidly induced in several cell types (namely neurons and radial glia) in response to various stimuli, such as cytokines and pro-inflammatory molecules [30]. COX-2 has been implicated in the conversion of a minor proportion of AEA and 2-AG to prostaglandin ethanolamides (PG-EAs) [26] and prostaglandin glycerol esters [31], respectively—both these types of prostaglandins can contribute to inflammatory responses [30]. Other prostaglandins that are produced from AA by COX-2, prostaglandin E_2_ (PGE_2_), and prostaglandin F_2_α (PGF_2α_) have been found to be neurotoxic [32,33]. The suppression of MAGL activity (and thus reduction in levels of AA) has shown neuroprotective effects in mice [34]. In a rat model of epilepsy, the levels of 2-AG and CB1 receptor protein expression were significantly upregulated in the hippocampi after induced epileptic seizures [35], but an increase of 2-AG in a mouse model before the inducement of seizures was reported to reduce the subsequent incidence of seizures [36]; this might indicate that the timing of an upregulation of 2-AG in the brain is critical for its seizure suppression effect. The COX-2 levels have been found to be significantly increased in the brains of patients with epilepsy [37] and in animals that experience prolonged seizures [38], which strongly suggest that neuroinflammation and epilepsy are indeed linked.

The Cytochrome P450 (CYP) family is another group of enzymes that metabolize endocannabinoids. The CYP enzymes are differently distributed in each tissue, and they differ across species as well as between individuals. The CYP enzymes have been studied (extensively from liver tissue or microsomes) for their ability to metabolize therapeutic drugs, inactivating many, but activating some for their therapeutic effect [39]. Given the extensive nature of the CYP pathways [28,29], we will only discuss some that are the most relevant to this review. CYP3A4, a CYP enzyme that is present in the human liver and brain [40,41], converts AA into epoxyeicosatrienoic acids (EETs) and hydroxyeicosatetraenoic acids (HETEs). CYP3A4 and CYP2C19 can also catalyze the conversion of AEA into the ethanolamide variants of EETs and HETEs, i.e., epoxyeicosatrienoic acid-ethanolamides (EET-EAs) and hydroxyeicosatetraenoic acid-ethanolamides (HETE-EAs)., respectively [42]. EETs have been found to be anti-inflammatory, while HETEs have been reported to be pro-inflammatory [43,44,45]. The EET-EAs and HETE-EAs interact with the CB1 and CB2 receptors with different affinities than AEA and 2-AG, e.g., in rodents, 5,6-EET-EA binds 1000-fold more strongly with CB2R than AEA [46], while 20-HETE-EA and 14,15-EET-EA bind weakly to CB1R [47]. 2-AG has been reported to be metabolized by CYP2J2 to form 2-11,12-epoxyeicosatrienoic glycerol (EET-G) and 2-14,15-EET-G [48]. In a rat model, 2-14,15-EET-G was detected in the brain. Both EET-Gs have a high affinity for the CB receptors, in particular CB1R [49]. Thus, alterations in CYP activity could affect the downstream activation of the endocannabinoid receptors.

The lipoxygenase (LOX) enzymes constitute another pathway by which the endocannabinoids and other fatty acids are metabolized [50]. The 5-LOX enzyme (expressed on neurons) catalyzes the conversion of AA into leukotriene A4. Leukotriene A4 (LTA_4_) is quickly converted to other leukotrienes, such as LTB_4_ and cysteinyl leukotrienes (Cys-LTs, i.e., LTC_4_, LTD_4_, and LTE_4_) [27,50]. LTD_4_ has been found to increase the blood-brain barrier dysfunction [51], which is associated with neuroinflammation and epileptogenesis [52]. Yu et al. reported that the exposure of mice microglial Cys-LT1 and Cys-LT2 receptors to LTD_4_ resulted in microglial phagocytosis and the secretion of the pro-inflammatory IL-1β [53]. In summary, much of the research on all of these eicosanoids (collective term for the enzymatic metabolites of polyunsaturated fatty acids, such as AA, 2-AG, and AEA) indicates that the ECS is deeply involved in neuronal activity and the regulation of neuroinflammation. However, until further research is undertaken to elucidate the signaling pathways involved in detail, particularly in the human brain, the question as to whether epilepsy is a cause or consequence of dysfunction in the ECS remains unanswered.

## 3. Cannabinoids Are an Emerging Treatment for Drug-Resistant Epilepsy, Especially in Children

Children are most detrimentally affected by refractory epilepsy, as they are not only at increased risk of death due to seizure-related accidents and respiratory infections, but they also have impaired neurodevelopment that might be caused by underlying epileptogenic processes, independent of the seizures themselves [54]. Refractory epilepsy affects approximately 10–20% of minors with epilepsy, and it can have devastating implications for their education, cognitive function, and social activities [55]. Families of affected epileptic individuals have advocated for alternative solutions, such as the use of medical cannabis, as many cases of refractory epilepsy prove to be resistant to current treatment options [3]. A noteworthy case study, popularized in a 2013 television documentary, is Charlotte Figi, a five-year-old girl who was having about 50 drug-resistant epileptic seizures per day. After Charlotte’s mother administered to her (in conjunction with Charlotte’s antiepileptic drug regimen) an oil that was derived from a particular strain of the cannabis sativa plant, her seizures decreased significantly to 2–3 nocturnal convulsions per month and her development improved to the point where she downsized her drug regimen; the term ‘Charlotte’s Web’ was later coined for Charlotte’s cannabis oil mix [56].

Cannabis plant extracts have been used for treating neurological disorders, such as epilepsy, since ancient times [57]. Phytocannabinoids (more commonly known as cannabinoids) are the active molecules present in cannabis, and they have reported therapeutic effects for managing a wide range of medical conditions, such as chronic pain, nausea, and multiple sclerosis [58]. However, scientific evidence has been limited to support the many claims about cannabis’ health benefits (e.g., for improving mental health), due to its status as an illegal drug in most countries and governmental restrictions to researching its effects [58]. The cautionary approach to the prescription of medical cannabis within the medical and scientific community [59] has started to contrast with the increasing community interest in cannabinoid products for epilepsy [56]. This surge in interest has been shaping a recent shift in government policy in some countries, like Uruguay and Canada, which have legalized recreational and medicinal cannabis in 2013 and 2018, respectively [60,61], while Israel and a majority of states (currently 37) in the USA have legalized cannabis for medical use [62]. Luxembourg is proposing to officially become the first member of the European Union to fully legalize cannabis in the near future, with Mexico and New Zealand also considering legalization within the next few years [60]. In Australia, the Therapeutic Goods Administration (TGA) currently allows for strict, limited prescription of medical cannabis by registered medical practitioners [63], and, as of September 2019, the Australian Capital Territory has legalized the individual possession and cultivation of small amounts of cannabis [64]. The gradual increase in the acceptance of pharmaceutical cannabinoids is leading to a corresponding rise in cannabis research, in an effort by the growing cannabis industry and pharmaceutical companies to better understand the underlying mechanisms by which cannabinoid products affect pathologies and, thus, legitimize their usage.

**Figure 2:**
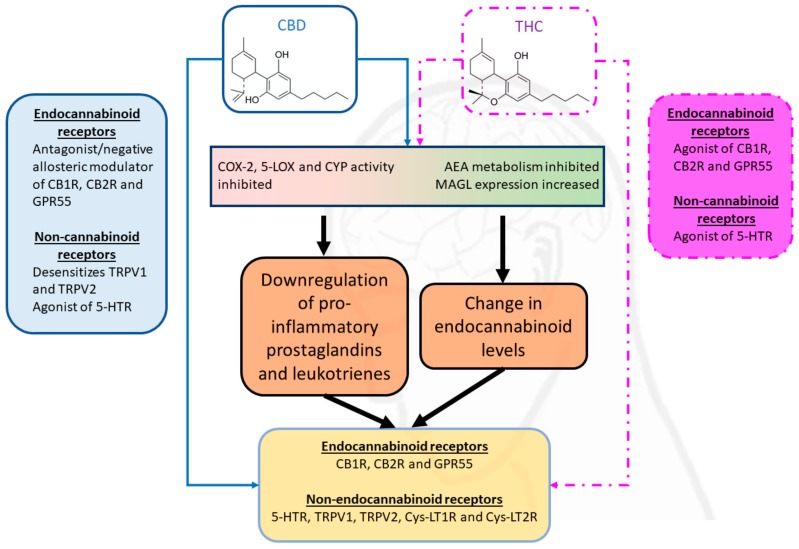
Interactions of the two major cannabinoids cannabidiol (CBD) and tetrahydrocannabinol (THC) with factors of epileptogenesis. This diagram summarizes the current evidence that cannabinoids can alter the levels of endocannabinoids and their by-products, as well as interact directly with many receptors (only some of which are shown in this diagram) in the brain.

Delta-9-tetrahydrocannabinol (Δ9-THC or THC) and cannabidiol (CBD) are the two cannabinoids that have been the most studied so far. THC is the primary psychoactive cannabinoid that confers the characteristic psychotropic effects that are associated with cannabis consumption, such as analgesia, euphoria, sensory alterations, short-term memory loss, appetite stimulation, and cognition impairments [65]. CBD shares many structural similarities with THC (as shown in Figure 2 below), but it is not psychoactive. CBD has been demonstrated to act on many parts of the CNS in animal and human models of epilepsy in ways that suggest anticonvulsant and neuroprotective effects [66,67,68,69,70]. Therefore, cannabinoids could be viable alternatives to standard drug treatments for the management of epilepsy.

However, chronic use of cannabis has also been associated with pathological and behavioral toxicity (primarily associated with THC), which varies with individuals as well as over time [71,72], which makes the effects of cannabis treatment unpredictable with current methodology. For example, cannabinoids have the potential to interfere with the neurodevelopment of children and adolescents, with their wide-ranging effects on the CNS and particularly the ECS [12]. Given the well-founded concerns regarding the long-term safety of THC, CBD has recently attracted the favor of the medical community as the most promising therapeutic cannabinoid due to its lack of psychoactive effects, relative safety [73], and purported benefits. According to the recent findings of three rigorous placebo-controlled randomized trials [74,75,76], CBD-based pharmaceutical formulations show promise as effective anticonvulsants, especially in treating refractory epilepsy [77]. In these trials, the participants’ pre-existing treatment regime (including medications and/or interventions for epilepsy, such as a ketogenic diet and vagus nerve stimulation) remained unchanged throughout. Two of the trials focused on the treatment of Lennox-Gastaut syndrome, while the third one selected patients that were affected by Dravet syndrome; both neurological disorders are severe forms of refractory epilepsy. All of the trials had minors participating (two to 55 years of age for the Lennox-Gastaut syndrome trials and two to 18 for the Dravet syndrome trial), involved the same oral formulation of 98% CBD (Epidiolex^®^ by GW Pharmaceuticals) and had a similar structure in terms of duration, i.e., they began with a four-week baseline period followed by a two-week escalation phase and a 12-week maintenance period (totaling a treatment period of 14 weeks). A maximum dose of 20 mg of CBD per kg of the participant per day was administered to the patients in all three trials, while one trial had an additional arm of 10 mg/kg/day [74,75,76]. While all three trials reported a significantly greater than 50% convulsive seizure reduction in participants on CBD when compared to those on placebo, there were also more adverse events described in participants on 20 mg/kg/day of CBD (about 90%) than on placebo (approximately 72%) [74,75,76]. Somnolence was the most common detrimental side-effect, followed by diarrhea and decreased appetite. Elevated liver transaminase enzyme levels (an indicator of liver toxicity) were more than three times the normal levels in patients on valproate [74]; the elevated levels were also more frequent in patients on CBD than placebo when co-administered with valproate (19% vs. 5%) [76]. The elevations tended to appear early in treatment and reversed spontaneously or following dose reduction or discontinuation of valproate or cannabidiol. The elevations suggest a potentially harmful interaction between valproate and CBD, even though there was no lasting liver distress [76]. Withdrawals from trials due to adverse events were higher in those taking CBD (7.2%–15%) than placebo (approximately 1.5%) [74,75,76,77,78]. The interaction of CBD with the metabolism of other drugs has been purported to be responsible for these adverse effects due to the trials not assessing the efficacy of CBD without co-administration of other AEDs, and that the reported side-effects are characteristic of many AEDs. CBD is oxidized in the liver by a range of CYP isoenzymes, particularly CYP3A4, CYP2C9, and CYP2C19 [79,80]; CYP2C19 is the principal enzyme initiating the breakdown of CBD to 7-hydroxy-CBD, which is then further metabolized to 7-carboxy-CBD by CYP3A4 [81]. As previously mentioned, the CYP enzymes are involved in the metabolism of xenobiotics, such as AEDs (e.g., clobazam) and their metabolites (e.g., N-desmethylclobazam) [82], and their polymorphic variants (which varies across different individuals) may be responsible for the differences in treatment response among individuals. CBD is theorized to bind to the enzymes’ active sites [80], hence inhibiting the catalysis of AEDs and increasing their half-life in the participants’ bodies, leading to a corresponding increase in antiseizure activity, elevated liver enzyme levels, and sedative side-effects [82]. Elevated plasma levels of N-desmethylclobazam in patients concomitantly treated with cannabidiol seemingly support this theory, but more recent studies have found that the N-desmethylclobazam plasma levels increased in patients that were also treated with CBD were linked with increased sedation, but were not correlated with seizure reduction in patients [81,83,84]. This elevation did not occur in the presence of another AED, stiripentol [85], and reinforces the notion that the increase in N-desmethylclobazam is due to CBD’s metabolism by CYP2C19, as stiripentol inhibits CYP3A4 and CYP2C19 [81]. These types of interactions of CBD with xenobiotics can mask the true independent effects and side-effects of CBD on epileptic patients [73] and are an example of why a better understanding of the mechanisms of cannabinoid action by experimentation could greatly help to improve the quality of cannabinoid-based treatment in patients.

As there are about 120 cannabinoids currently characterized in cannabis plant variants [86], the expanding field of cannabinoid research has so far not been able to fully determine the effects of each cannabinoid on the human body, and their interactions with each other and other drugs [87]. As highlighted by the interaction of CBD with the CYP isoenzymes, a comprehensive understanding of cannabinoid pharmacokinetics is crucial in properly identifying the onset, magnitude, and duration of both the negative and positive effects of cannabinoid exposure. High quality scientific data on the safety of long-term exposure to cannabinoid-based medicines in children is needed for effective treatment and regulation [71], since most laboratory studies so far have only looked at the short-term effects of cannabinoids in animal studies and/or on cultured cells [68], and rigorously controlled clinical trials have thus far not administered cannabidiol for longer than 14 weeks [73,74,75,76]. Even though many clinical trials extend into open-label, longitudinal studies in which trial participants can enroll, most of the clinical studies partly rely on self-reporting and/or reporting from parents/guardians to assess the effectiveness of the administered treatment; this type of information can sometimes be unreliable, biased, and inadequate for the measurable monitoring of therapy [77,88], but it can be supported by correlation with measured analysis of participants’ samples (as demonstrated by the monitoring of the N-desmethylclobazam levels mentioned previously) [81,82].

### Cannabinoids Interact with the Endocannabinoid System

The effects of cannabinoids on epilepsy may be attributed to their interactions with the ECS (summarized in Figure 2), ion channels, and other mediators of neuroinflammation.

THC is a partial agonist of CB1 and CB2 receptors and an agonist of GPR55, while CBD is an antagonist/negative allosteric modulator of the CB1, CB2 and GPR55 receptors [89,90,91,92], which could partly explain how CBD regulates the psychotropic activity of THC when simultaneously administered [93]. While CBD binds weakly to CB1 and CB2 at therapeutic doses [94], its antagonism of GPR55 represses intracellular calcium release and reins in the characteristic neuronal hyperactivation of epilepsy [92,95]. The administration of CBD has been reported to be correlated with an increase in the serum levels of AEA in treated schizophrenic patients vs. placebo-control patients [96]; this could potentially be a pathway by which CBD exerts a beneficial effect, because it helps to counteract the lower levels of AEA that were measured in the cerebrospinal fluid of epileptic patients [24]. The mechanism by which CBD increases AEA levels has not been fully elucidated and it requires further investigation. Elmes et al. reported that, in humans, this effect might be due to THC and CBD preferentially binding to the fatty acid binding proteins on which AEA depends to be transported intracellularly to be catalyzed by intracellular FAAH [97]. Of note, this signaling contrasts with the cannabinoids’ inhibition of FAAH activity in rodents [97,98], demonstrating that differences in xenobiotics metabolism between species can limit the utility of animal models in cannabinoid research. An upregulation of *Magl* gene (gene that encodes for the MAGL enzyme) expression in the hypothalamus of rats that were treated with 10 mg/kg THC has been observed [99], which reinforces the notion that cannabinoids can modulate endocannabinoid tone in the brain. Cannabinoids, including THC and CBD, have been found to inhibit COX-2 activity and, hence, reduce the production of pro-inflammatory prostaglandins, which could be another means by which cannabinoids increase the levels of the endocannabinoids and exert an indirect anti-inflammatory and subsequent anti-epileptic activity [100,101]. CBD’s inhibition of the CYP isoenzymes in the brain could, in turn, modulate the synthesis of EETs, EET-EAs, and HETE-EAs. Therefore, we posit that CBD might exert an indirect action on the activity of these endocannabinoid receptors via the upregulation/downregulation of downstream eicosanoids even though CBD does not itself act strongly on CB1 and CB2 receptors [40]; for example, Bornheim et al. found that CBD inhibited the CYP-driven formation of some AEA metabolites in mice [102], while Arnold et al. reported that THC and CBD inhibited the production of EET-EAs by cardiac CYP2J2 [103]. Additionally, CBD decreased the activity and metabolites of 5-LOX in human tumor cells [104]. As some targeted inhibitors of Cys-LT synthesis have been demonstrated to significantly attenuate seizures in treated mice (compared to untreated mice) [105,106] and in epileptic patients [107], this could be an avenue by which CBD exerts an anti-seizure effect, but requires further validation.

Interestingly, cannabinoids, such as CBD and cannabidivarin (CBDV), have been shown to desensitize the non-cannabinoid Transient Receptor Potential Vanilloid 1 (TRPV1) (that can be activated by AEA [108]) and TRPV2 ion channels preventing extracellular calcium ion secretion and downregulating neuronal hyperexcitability (an important factor of epileptogenesis), which suggests another potential anticonvulsant mechanism [98,109]. CBD has been reported to partially enhance microglial phagocytosis in rodent microglia via the activation of TRPV1 and probably the TRPV2 receptor channel of the microglial cells [8]; Hassan et al. cautioned that increasing microglial phagocytosis might not be a positive strategy for combating neuroinflammation, but their results have yet to be replicated in human cells. As mentioned beforehand, the cannabinoids may indeed exert their effects differently between species. Another example of non-cannabinoid interaction is THC and CBD’s agonistic actions on the serotonin (5-hydroxytryptamine) receptors (5-HTR), which are highly involved in many of the processes related to cannabis’s actions (e.g., relief of anxiety and pain) and neuronal electrochemical activity [110,111,112]. CBD has been shown to inhibit the equilibrative nucleoside transporter (ENT1) that is involved in the synaptic uptake of adenosine, thereby increasing extracellular adenosine. The increased levels of extracellular adenosine, in turn, decrease neuronal hyperexcitability and neurotransmission [113,114,115]. Another potential route of antiseizure activity for CBD could be its inhibition of voltage-dependent anion selective channel protein (VDAC1) channel conductance, which could have an immunosuppressive effect and, hence, downregulate neuroinflammation [116].

THC might have a synergistic effect with CBD (i.e., THC improves CBD’s medicinal properties while CBD attenuates THC’s psychotropic effects due to its antagonism of CB1 and CB2 receptors [87,117]) and could be therapeutically used [118,119], but THC’s psychoactive effects and strong interaction with endocannabinoid receptors (such as CB1R) can be detrimental in a therapeutic context, especially in minors. Endocannabinoids and cannabinoid receptors are both present in the brain since early developmental periods and are immature until adulthood [15,120]; in rats, the density of CB1 receptors has been shown to increase during normal development, peaking in adolescence, before decreasing to adult values [121,122]. THC’s interaction with CB1R has been hypothesized to cause alterations in the density of CB1R in the human brain [123]. Indeed, some studies have demonstrated potentially detrimental alterations in the brain structure/function (particularly in the cortical region) of adult and adolescent rodents [124,125,126], as well as humans [127,128] consuming cannabis when compared to cannabis-free controls. However, other experiments investigating changes in the brain morphology of cannabis users have reported no significant difference [129] or even contradictory findings; for example, one study found thinner brain cortices in adolescent/young adult cannabis users [130], while another study reported increased cortical thickness in adolescent cannabis users [131], when compared to non-using controls. This lack of definitive evidence regarding the long-term effects of cannabinoids on the human brain reinforces the need for more accurate measurement of the interplay between the ECS and cannabinoids.

## 4. Biomarkers for Determining Neuroinflammation and Monitoring Cannabinoid Effects

The monitoring of cannabinoid therapy in the successful management of epilepsy/neuroinflammation could potentially be achieved by the development of biomarkers sampled from easily obtainable biological fluids (e.g., blood, saliva) other than directly from the brain due to the circulation of cytokines, eicosanoids and cannabinoids in the human body [18]. Biomarkers are molecules that vary with features of disease or treatment, and their measurement therefore provides information regarding disease progression and/or the effectiveness of treatment. The range of biomolecules that can be screened as biomarkers is wide, and it includes RNA (especially messenger RNA and micro RNA), proteins, cytokines, endocannabinoids, prostaglandins, metabolites, and signaling molecules (e.g., calcium ions). Extracellular vesicles, specifically exosomes, have been found to contain a range of biological substances, and they are therefore an attractive source of potential biomarkers. Exosomes are nanometer-sized, membrane-bound bodies that are central to cell-cell communication. Exosomes are secreted via the exocytosis of multivesicular bodies from cells/tissues to circulate into biological fluids (such as milk, saliva, urine, and blood), can cross the blood-brain barrier [132,133], and can contain a range of factors including RNA, lipids, and proteins. They have been demonstrated to transfer functional genetic material (such as mRNA and miRNA) to cells, which is subsequently expressed as protein in the target cells [134] and signaling molecules (such as eicosanoids) to activate inflammatory pathways in recipient cells [135,136,137,138].

A single (or similar type of) biomarker is unlikely to provide enough information to track disease progression and/or treatment to assess and improve the effectiveness of cannabinoid treatment in human epileptic patients, as demonstrated by the measurement of CYP enzyme activity levels that provided an incomplete picture of human CBD metabolism. Several groups have researched biomarkers of epilepsy and neuroinflammation. For example, Persike et al. and Kan et al. both found that the brain hippocampi of epileptic patients had altered protein profiles when compared to seizure-free patients, and it is possible that these protein changes could also be detected in circulating biofluids, as is the case for cancer biomarkers that have been detected in patients’ blood [139]. In addition, Kan et al. reported an upregulation in inflammatory mediators (such as IL-10 and IL-1β) in epileptic patients [140], while Persike et al. demonstrated that there was differential expression of proteins involved in neuronal health, metabolic activity, and oxidative stress in post-mortem epileptic human hippocampi [141]. Circulating (and therefore accessible) biomarkers of treatment response have also been identified in epilepsy. Wang et al. found alterations in the serum miRNA profile of drug-resistant epileptic patients when compared to that of drug-responsive epileptic patients, but the ethnical homogeneity of patients studied and the inability to fully determine the downstream effects of the miRNAs limited the usefulness of their findings [142]. Elevated COX-2 activity that was indicative of neuroinflammation was detected in epileptic mice hippocampi by an associated increase in the PGE2 levels, as shown by Takemiya et al. [143]. Wolfe and Mamer reported increased levels of PGF2α in the cerebrospinal fluids of human epileptic patients when compared to patients with no seizures [144]. Yan et al. have reported altered miRNA profiles in the plasma exosomes of epileptic patients as compared to healthy control patients; however, the impact of their findings is limited by their single source of exosomes (i.e., blood plasma) and uncertainty about the exosomes’ points of origin, which makes the determination of the miRNAs’ targets and their actual physiological effects difficult [145].

Another technique for measuring neuroinflammation is by imaging biomarkers of the neuroimmune system. Magnetic Resonance Spectroscopy (MRS) is an imaging technique that facilitates the quantitative measurement of metabolites in localized brain volumes. The neurometabolite myo-inositol is a sensitive biomarker for the neuroimmune system. It is predominately located in astrocytes and it increases with inflammation, but lacks specificity. Positron Emission Tomography (PET) imaging of the 18-kDa translocator protein (TSPO) provides a quantitative measure of a biomarker expressed in neuroimmune cells (such as microglia). However, it can lead to false positives, as it can be elevated in the absence of neuroimmune activation. Using multimodal techniques (MRS and PET) might allow for the measurement of the neuroinflammatory effect of cannabidiols as myo-inositol and TSPO measure related but distinct immune processes [146].

The cannabinoids and their metabolites can be detected and measured in a variety of biological fluids, like blood plasma [147], saliva, and urine [148] to evaluate their intake and study their metabolism [149]. Therefore, a suite of different types of epileptic and neuroinflammatory biomarkers (especially the downstream mediators, such as IL-1β and COX-2 products) in conjunction with biomarkers of cannabinoid treatment (e.g., cannabinoids and their metabolites) could help to paint a more complete picture of the cannabinoids’ metabolic pathways, their toxicity, and their modes of action on the neuroinflammatory pathways. All of this complementary information could be potentially used to improve the delivery of cannabinoid-based treatment, and even help synthesize artificial cannabinoids or ECS inhibitors that are more selective, have greater potency, and cause less undesirable side-effects than the cannabis plant extracts [150,151].

## 5. Conclusions

In the course of this review, we discussed the strong evidence linking epilepsy, the ECS, and cannabinoids. The ECS’s vital role in neuronal health and inflammation has been demonstrated to be a major actor in epileptogenesis via a multitude of signaling pathways. Cannabinoids (particularly CBD) show promise as a potent antiseizure treatment, but the promiscuous binding affinity of the cannabinoids for numerous receptors makes the task of identifying their beneficial and detrimental mechanisms of action challenging. The development of multiple types of biomarkers could advance the characterization of the crucial signaling pathways and pave the way for better cannabinoid-based therapies.

As of the time of writing, the application of cannabis for medicinal purposes is predicted to rapidly expand in the near future [60]. The CBD-containing drug Epidiolex was approved for the treatment of epileptic seizures by the USA’s Food and Drug Administration (FDA) in June 2018 [152]. Cannabinoid treatment has also been found to be successful in a few case studies and small clinical trials treating other neurological disorders, such as tic disorders [153] and autism spectrum disorders [154,155], whereby the cannabinoids’ therapeutic mechanisms of action in human patients are largely unknown. Research regarding the physiological effects of minor cannabinoids (e.g., CBDV and cannabigerol) is currently lacking [109,156] and could become more urgent, as new cannabis strains may be created that produce higher concentrations of these minor cannabinoids. Recently, GW Pharmaceuticals conducted a Phase 2a proof of concept study of their proprietary CBDV compound in adult patients with focal seizures, which did not meet its primary endpoint—in the trial’s preliminary results, active and placebo cohorts both showed similar reductions in focal seizures. The extent of this high placebo response is substantially greater than that seen in published studies of other treatments in similar patient populations and the potentials reasons for this result are still being investigated [157]. A key issue with cannabinoid treatment, as recently highlighted, is the dosing and administration route of treatment. The uptake of orally administered CBD has been found to be affected by the level of fat in meals taken close to the time of medication [158]: high-fat meal intakes were positively correlated with a four-fold increase in the blood level of CBD when compared to fasting conditions. Therefore, food intake could be a considerable source of variability in CBD bioavailability and it will most probably need to be taken into consideration when prescribing cannabinoids. Other forms of drug administration are being explored; for example, Zynerba Pharmaceuticals are trialing the administration of CBD via a proprietary transdermal patch. Exosomal delivery of cannabinoids could improve the efficacy of drugs to patients, since oral administration of CBD can lead to limitations in safety and efficacy that include gastrointestinal side effects, like nausea, vomiting and diarrhea, low bioavailability, inconsistent plasma levels (which vary widely across patients), and significant first-pass liver metabolism (first-pass liver metabolism refers to the liver processing drugs ingested through the gastrointestinal system, such as through oral or oral-mucosal delivery methods, only allowing for a small amount of drug to be absorbed into the circulatory system and delivered to the brain) [159]. To bypass these limitations, exosomal delivery could be a viable alternative to oral administration of CBD. Indeed, Zhuang et al.’s intranasal delivery of xenobiotic-loaded exosomes to the neural microglia of live mice encouragingly showed a targeted mode of exosomal drug delivery in vivo [160]. Exosomes that were engineered to express the neuron-specific rabies viral glycoprotein (which improves their selective targeting of brain cells) were recently demonstrated to cross the blood-barrier barrier in vivo [132]; this could therefore be a promising avenue for delivering cannabinoids on-target in the future.

The legalization of cannabis also brings up other concerns in the domain of public health, since it may promote the casual consumption of cannabis in the general population [161]. The crucial role played by the ECS in mammalian fertility [162,163] and pregnancy has been shown to be disrupted by the presence of cannabinoids—a disruption of the maternal ECS could lead to complications of pregnancy (such as miscarriage) and the cannabinoids themselves could negatively impact the development of the embryo/infant as the lipophilic molecules can easily cross the placental barrier and disturb the proper functioning of the embryonic/fetal ECS [164,165]. Such upcoming issues bring the current gaps in our knowledge of cannabinoids to light and call for an increase in research on this matter to better regulate and legislate the usage of cannabis.

## Abbreviations

2-AG	2-arachidonoylglycerol
5-HTR	5-hydroxytryptamine receptor
AA	Arachidonic acid
AEA	N-arachidonoyl-ethanolamine or anandamide
AEDs	Anti-epileptic drugs
CBD	Cannabidiol
CBDV	Cannabidivarin
Cys-LT	Cysteinyl leukotriene
CNS	Central nervous system
COX	Cyclooxygenase
CYP	Cytochrome P450
EA	Ethanolamide
ECS	Endocannabinoid system
EET	Epoxyeicosatrienoic acid
ENT1	Equilibrative nucleoside transporter
FAAH	Fatty acid amide hydrolase
FDA	Food and Drug Administration
GPR	G-protein coupled receptor
HETE	Hydroxyeicosatetraenoic acid
HPETE	5-hydroperoxyeicosatetraenoic acid
IL-1β	Interleukin-1β
LOX	Lipoxygenase
LT	Leukotriene
MAGL	Monoacylglycerol lipase
mRNA	Messenger ribonucleic acid
miRNA	Micro ribonucleic acid
MRS	Magnetic Resonance Spectroscopy
PET	Positron Emission Tomography
PG	Prostaglandin
PGH_2_-G	Prostaglandin H_2_-glycerol ester
RNA	Ribonucleic acid
TGA	Therapeutic Goods Administration
TNF-α	Tumor necrosis factor-α
Δ9-THC or THC	Delta-9 tetrahydrocannabinol
TRPV	Transient Receptor Potential Vanilloid
TSPO	Translocator protein
VDAC	Voltage-dependent anion selective channel protein
WHO	World Health Organization
